# Health outcomes related to the provision of free, tangible goods: A systematic review

**DOI:** 10.1371/journal.pone.0213845

**Published:** 2019-03-20

**Authors:** Nav Persaud, Liane Steiner, Hannah Woods, Tatiana Aratangy, Susitha Wanigaratne, Jane Polsky, Stephen Hwang, Gurleen Chahal, Andrew Pinto

**Affiliations:** 1 Centre for Urban Health Solutions, St. Michael’s Hospital, Toronto, Canada; 2 Department of Family and Community Medicine, St. Michael’s Hospital, Toronto, Canada; 3 Department of Family and Community Medicine, Faculty of Medicine, University of Toronto, Toronto, Canada; 4 Division of General Internal Medicine, University of Toronto, Toronto, Canada; 5 The Upstream Lab, Centre for Urban Health Solutions, Li Ka Shing Knowledge Institute, St. Michael’s Hospital, Toronto, Canada; 6 Dalla Lana School of Public Health, University of Toronto, Toronto, Canada; Institute of Medical Psychology and Medical Sociology, GERMANY

## Abstract

**Background:**

Free provision of tangible goods that may improve health is one approach to addressing discrepancies in health outcomes related to income, yet it is unclear whether providing goods for free improves health. We systematically reviewed the literature that reported the association between the free provision of tangible goods and health outcomes.

**Methods:**

A search was performed for relevant literature in all languages from 1995-May 2017. Eligible studies were observational and experimental which had at least one tangible item provided for free and had at least one quantitative measure of health. Studies were excluded if the intervention was primarily a service and the free good was relatively unimportant; if the good was a medication; or if the data in a study was duplicated in another study. Covidence screening software was used to manage articles for two levels of screening. Data was extracted using an adaption of the Cochrane data collection template. Health outcomes, those that affect the quality or duration of life, are the outcomes of interest. The study was registered with PROSPERO (CRD42017069463).

**Findings:**

The initial search identified 3370 articles and 59 were included in the final set with a range of 20 to 252 246 participants. The risk of bias assessment revealed that overall, the studies were of medium to high quality. Among the studies included in this review, 80 health outcomes were statistically significant favouring the intervention, 19 health outcomes were statistically significant favouring the control, 141 health outcomes were not significant and significance was unknown for 28 health outcomes.

**Interpretation:**

The results of this systematic review provide evidence that free goods can improve health outcomes in certain circumstances, although there were important gaps and limitations in the existing literature.

## Introduction

Disparities in health along socioeconomic lines are well established: groups with lower income and socioeconomic position consistently experience worse health outcomes, including higher rates of mortality.[1, 2] One of many possible explanations for better health outcomes among those with higher socioeconomic status is that income allows greater access to tangible goods that can improve health, such as safe shelter, healthy foods, clean water, and essential medicines. Worse health outcomes among lower socioeconomic status groups may be explained by reduced access to education and child care, exposure to hazards such as air pollution or contaminated drinking water, exposure to violence, reduced access to health care services, or discrimination based on gender, ethnicity or other characteristics.[3, 4] Some of these potential alternative explanations may be indirectly related to access to tangible goods, such as water filtrations systems that can mitigate effects of contaminated water and medicines that may mitigate the effects of poor access to health care services. The importance of tangible goods has long been recognized through accounting for “non-cash” income, such as the value of housing provided by governments, and by defining poverty based on the cost of tangible goods (as in reference budgets that are baskets of goods and services that are considered necessary to reach an acceptable standard of living for an individual household within a given country, region or city) and essential services rather than based on relative income level.[5, 6]

If people lack a good that is required for their health and well-being, a simple response is to provide it for free. This approach appears to underpin many governmental and non-governmental programs routinely devote substantial resources to distributing goods to people in need.[7–9] Yet it is unclear whether providing goods for free promotes health. Free tangible goods may not be used as intended or at all: their positive health effects may not overcome other causes of poor health, or they may even cause unintended harm (e.g. providing safety equipment such as bicycle helmets could encourage risky behavior).[10] Providing people with free goods could complement other efforts to promote health, such as providing services like healthcare,[11] and providing a Basic Income.[12, 13] The receipt of free tangible goods could free up limited household income or resources that would otherwise be consumed in obtaining those goods and this additional disposable income may result in improved health.

We are not aware of any previous systematic effort in the existing scientific literature to assess whether providing free goods promotes health. We systematically reviewed the literature for studies that reported the association between the free provision of tangible goods and health outcomes.

## Methods

### Search strategy

A search strategy was developed in consultation with an information specialist. This systematic review was registered on PROSPERO (CRD42017069463, Aug 30 2017).

We defined “tangible goods” as a physical good or object that could be given to persons or families. We generated a list of items which were hypothesized to be distributed without charge to patients or study participants. The list of items was sent to several other researchers for feedback who had expertise in primary health care, social determinants of health, health economics, epidemiology, public health, homelessness, housing, refugee health, access to healthy food and income security. After feedback was received, a final list of key terms was created with all suggestions included (S1 File, Search strategy).

Key terms were searched in the following databases: EMBASE, MEDLINE, CINAHL, PsycINFO, Cochrane, ProQuest databases (others could include Applied Social Sciences Index and Abstracts (ASSIA), FRANCIS, International Bibliography of the Social Sciences (IBSS), PAIS International, ProQuest Family Health, ProQuest, Social Services Abstracts, Sociological Abstracts) in all languages from 1995-present. We also looked through trial registries. The search was conducted in June 2017.

### Inclusion criteria

Eligible studies were observational (e.g. case-control, cohort, before-after, pre-post or longitudinal), and experimental studies (e.g. randomized controlled trial), which had at least one tangible item provided free of cost to participants. Examples of free goods included transit passes, food boxes, infant goods, bicycle helmets, condoms, needles, and other drug paraphernalia. Studies had to have at least one quantitative measure of health. We understood “health” as the quality or duration of life. Although housing retention is not a health outcome, it was treated as such because housing is closely related to quality of life.[14]Included studies were also required to have a comparison or control group that allowed the effect of the free good to be measured. Studies published between January 1995 and May 2017 were eligible.

### Exclusion criteria

We excluded studies in which a service such as advice, health screening procedure or a diagnostic test was provided; if the intervention was primarily a service and the free good was relatively unimportant (e.g. giving participants a voucher for a health service); if the good was a medication (e.g. nicotine replacement, contraception, naloxone kits); or if the data in a study was duplicated in another study (duplicated data was defined as data from the same participant at the same timepoint).

### Screening

Covidence screening software [15] was used to manage articles while screening. In level one screening, all titles and abstracts were reviewed to determine if they met the inclusion criteria for the study. Level two consisted of screening the full text of articles to determine whether they met the inclusion criteria. Each article was appraised by two reviewers (LS and HW) for both levels and disagreements were discussed. If the reviewers did not come to a decision, a third investigator (NP) was consulted.

We attempted to include only one report of each health outcome. We excluded reports where both the outcomes and participants were the same as a study that was already included. We included reports where the participants and outcomes only partially overlapped between reports. If multiple reports included the same outcome for the same participants, we included that outcome only once.

### Extraction technique

Publication information, study characteristics, participant demographics, the health outcomes measured in the study and the quantitative results were extracted from each study by one reviewer using an adaption of the Cochrane data collection template. [16]

### Quality appraisal

The quality of each article was appraised by two individual reviewers using the Cochrane Risk of Bias assessment tool for randomized control trials [17] and ROBINS 1 assessment tool for non-randomized control trials. [18] The Cochrane Risk of Bias tool assesses seven potential sources of bias including random sequence generation, allocation concealment, blinding of participants, blinding of outcome assessments, incomplete outcome data, selective reporting, and funding source. [17] The ROBINS 1 tool also assesses seven potential sources of bias including bias due to confounding, bias in selection of participants into the study, bias in classification of interventions, bias due to deviations from intended interventions, bias due to missing data, bias in measurement of outcomes, and bias in selection of the reported results. [18] We did not exclude any studies based on the risk of bias assessment.

### Presentation of findings

We grouped studies based on the type of free good provided and the outcome reported.

## Results

### Literature search

**Fig 1:**
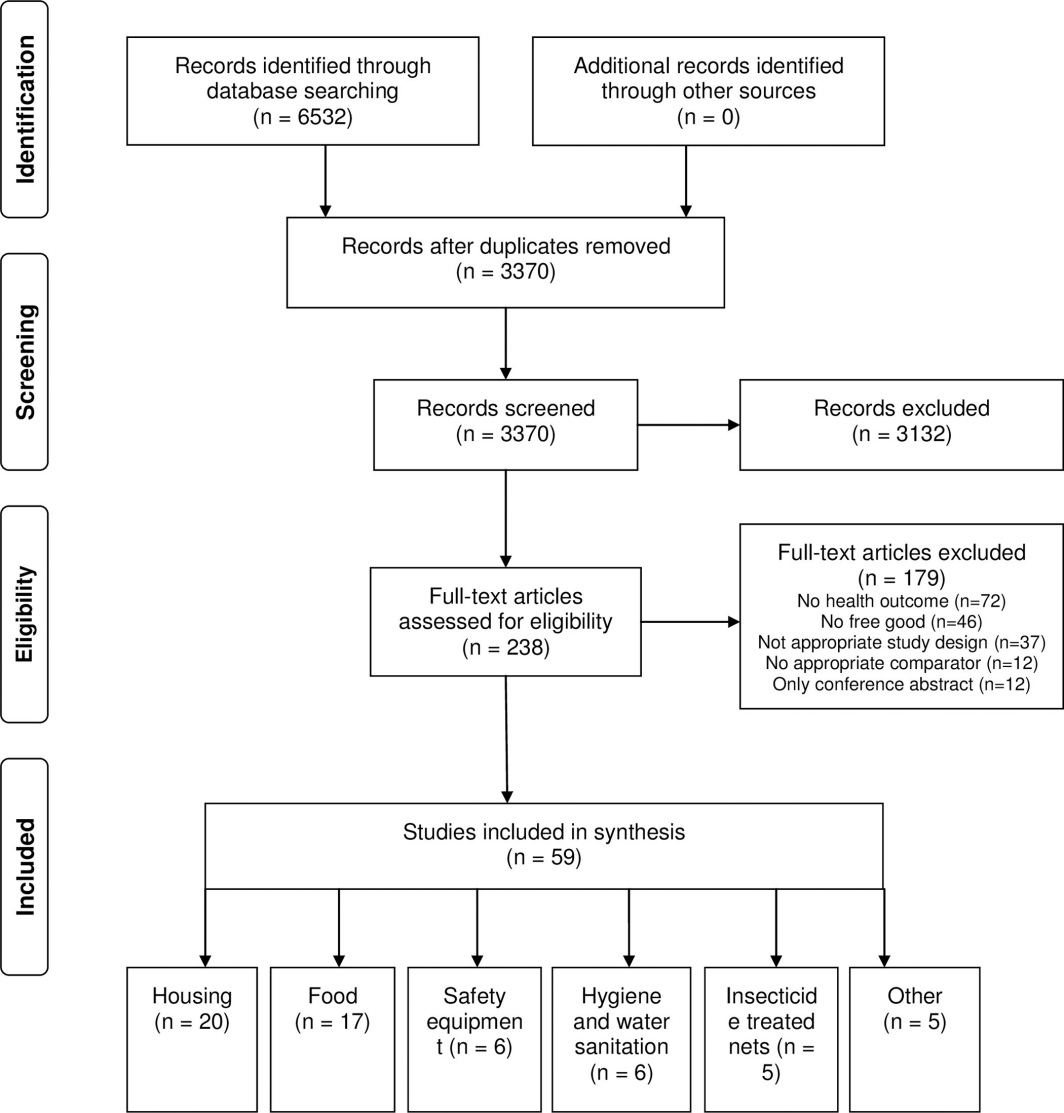
Flow diagram of study selection process. Adapted from PRISMA.[19].

The initial search identified 3370 articles of interest. In the first level of screening based on abstract review, 3132 articles were excluded, leaving 238 articles for full manuscript review. This second level of screening removed a further 179 articles yielding a final set of 59 articles which met full eligibility criteria (Fig 1).

### Study characteristics

The 59 included studies included a range of 20 to 252 246 participants with a median of 872·5. The length of the studies ranged from two to 180 months with a median of 15·5 months. Of the 59 articles, 29 were randomized controlled trials (RCTs) and 30 were observational studies.

Among the 59 included studies, 45 (76·3%) were from countries that are considered high income according to the 2016 World Bank Report.[20] These countries included the USA (20 studies), Canada (13 studies), United Kingdom (four studies), Norway (two studies), Israel (two studies), Ireland (one study), New Zealand (one study), Australia (one study), and France (one study).Fourteen studies (23·7%) were from countries considered low or medium income by the 2016 World Bank Report.[20] These countries included India (three studies), Cameroon (two studies), and one study each from Mexico, Colombia, Ukraine, Pakistan, Ghana, Kenya, Nigeria, China and Zanzibar.

Among the 59 included studies, the free goods provided were housing (20 studies), food (17 studies), safety equipment (six studies), insecticide treated nets (five studies), hygiene, and water sanitation (six studies) and miscellaneous (five studies).

### Risk of bias

**Fig 2:**
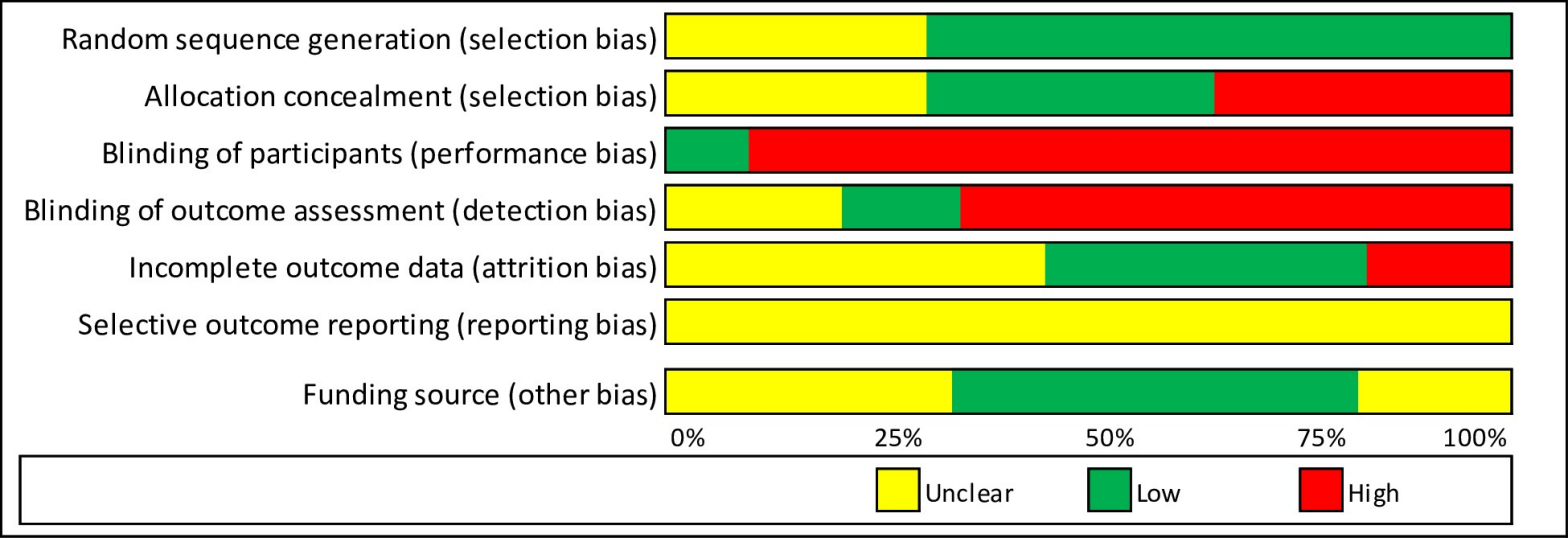
Cochrane risk of bias summary.

**Fig 3:**
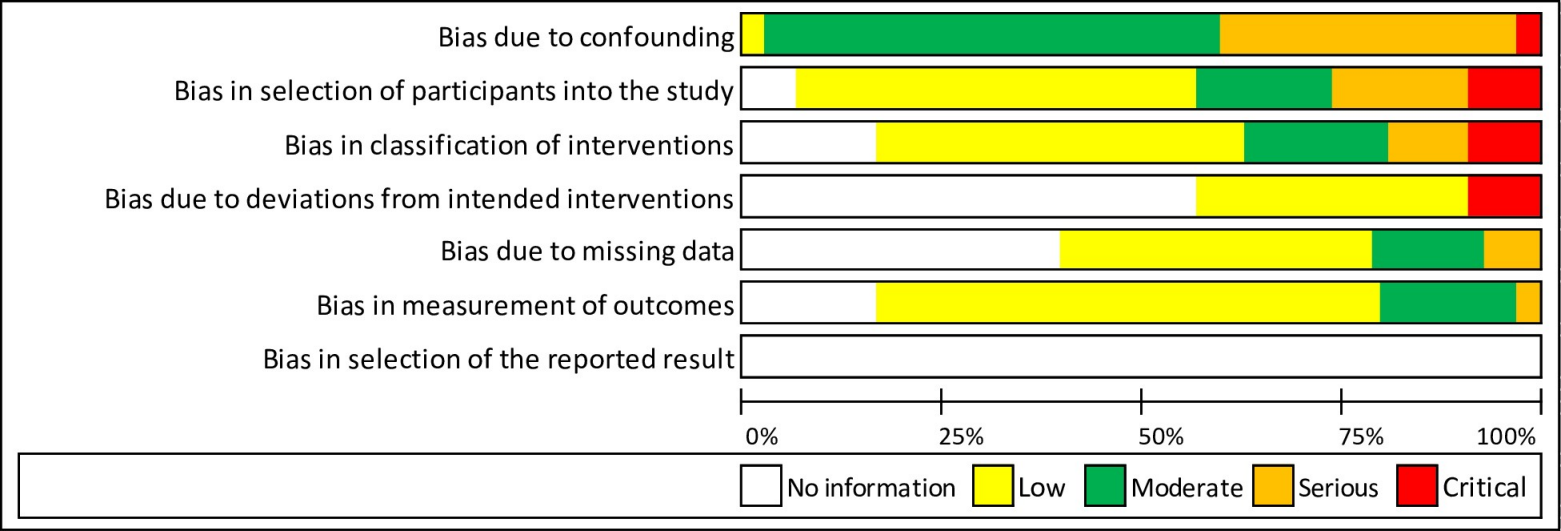
ROBINS 1 risk of bias summary.

Among the RCTs there were: no studies judged to be at a low risk of bias in all domains, one (3·4%) study was at a low or unknown risk of bias for all domains and 28 (96·6%) studies were at a high risk of bias in at least one domain (Fig 2). Among observational studies, there was: one (3·3%) study judged to be at a low risk of bias or no information in all domains, 11 (36·7%) studies at a low or moderate risk of bias or no information for all domains, 13 (43·3%) studies at serious risk of bias in at least one domain (but not at critical risk of bias in any domain), and five (16·7%) studies at critical risk of bias in at least one domain (Fig 3). Risk of bias assessment data is available as S1 Table, Cochrane risk of bias assessment for RCTs and S2 Table, ROBINS 1 risk of bias assessment for observational studies.

### Results by type of good

#### Housing

There were 24 940 participants in the 20 housing studies (there was some overlap in participants between studies; see the Methods section) (Table 1). All studies were conducted in either Canada (12 studies) or the USA (eight studies). Nineteen of these studies (95%) had a co-intervention, of which eighteen were “Housing First” programs. For example, in addition to housing, the intervention offered participants treatment for various addictions, mental health challenges and other social supports. [21] The primary reported outcomes in housing studies were stable housing (11 studies, 55%);substance use (10 studies, 50%); psychiatric symptoms or mental health,(eight studies, 40%); quality of life, including QoLI-20, community functioning (MCAS)and community integration (CIS-PHYS and CIS-PSYCH)(eight studies, 40%); health status, including BMI, waist circumference, physical health ailments and health assessments using EQ5D-VAS, and physical SF-12 assessment forms (six studies, 30%); food security (two studies, 10%); and death (one study, 5%). The study durations ranged from six months to 180 months. Housing studies reported a total of 114 outcomes (with duplicates removed), of which 42 were statistically significant, 62 were not significant, and significance was unknown for 10 outcomes. Of the 42statistically significant outcomes, 37 outcomes (from 15 different studies) favoured the intervention, and five outcomes (from two different studies) favoured the control.

**Table 1:** Characteristics of included housing studies (N = 20).

Study	Study type	Country	Participants	Intervention vs. Comparison	Co-intervention	Time	Health Outcome	Results*
Tsemberis 2004[21]	RCT	USA	225 Homeless adults with serious mental illness	Housing First vs treatment as usual	Participants in both groups had additional counseling and resources available	24 months	Residential stability	F _4,137_ = 27·7; p<0·001
							Alcohol use	F _4,136_ = 1·1; p = 0·35 *favours control*
							Drug use	F _4, 136_ = 0·98; p = 0·42 *favours control*
							Psychiatric symptoms	F _4, 137_ = 0·348; p = 0·85*favours control*
							Decrease in homeless status	F _4, 137_ = 10·1; p<0·001
Stefancic 2007[22]	RCT	USA	260 Homeless adults with serious mental illness *(originally assigned)*	Housing First vs treatment as usual	Participants in both groups had additional counseling and resources available	47 months	Housing retention at 20 months	Intervention: 103/ 209; Control:15/51*unknown significance*
Padgett 2011[23]	Qualitative interview	USA	83 Homeless adults with serious mental illness	Housing First vs treatment first	Participants in both groups had additional counseling and resources available	12 months	Substance use during the program *(number of people)*	X^2^ = 8·458;df = 1; p = 0·004
Jacob 2013[24]	Observational	USA	11680 Children in public housing with their family	Housing voucher vs no housing voucher	NR	NR	Deaths from disease	OR 0·91 (95%CI: 0·30–2·22); p = 0·84*favours control*
							Deaths by homicide	OR 1·07 (95%CI: 0·6,1·79); p = 0·81*favours control*
							Accidental deaths	OR 2·13 (95%CI: 0·66–5·99); p = 0·19*favours control*
Montgomery 2013[25]	Observational	USA	177 Homeless veterans with mental illness	Housing First vs treatment as usual	Participants in both groups had additional counseling and resources available	12 months	Housing first: *using logic regression model estimating relationship between intervention and housing stability*	OR 8·332; p = 0·023
Patterson 2013[26]	RCT	Canada	497 Homeless adults with serious mental illness in Vancouver	Housing First vs treatment as usual	Participants in both groups had additional counseling and resources available	12 months	QOL moderate needs	Intervention: baseline 72·2 (SD: 21·6); follow up 91·3 (SD: 20·6); Control: baseline 72·8 (SD: 23·3); follow up 85·7 (SD: 23·2); p = 0·095*favours control*
Palepu 2013[27]	Parallel RCT	Canada	497 homeless adults with serious mental illness in Vancouver	Housing First vs treatment as usual	Participants in both groups had additional counseling and resources available	12 months	Housing first vs treatment as usual association with residential stability	Adjusted incidence rate ratio 4·05 (95% CI: 2·95–5·56)
							Days in stable residence for people with substance dependence	Intervention: 255·9 (SD: 103·8); Control: 68·1 (SD: 108)*favours control*
							Days in stable residence for people without substance dependence	Intervention: 254·3 (SD:113·1); Control: 72·3 (SD:114·7)*favours control*
Bean 2013[28]	Longitudinal	USA	20 medically vulnerable and homeless participants who received housing and peer support by Project H3	Baseline *(at the day of move-in to housing)* vs follow up *(12 months after move-in)*	Participant received peer support, additional counseling and resources available	6 months	Physical-QOL,	Baseline: 3·08 (SD: 0·82); Follow-up: 3·51 (SD: 0·65); p = 0·008
							Psychological-QOL,	Baseline: 3·29 (SD: 0·87); Follow-up: 3·66 (SD: 0·72); p = 0·05
							Social Relationships,	Baseline: 3·19 (SD: 0·98); Follow-up: 3·62 (SD: 0·87); p = 0·05
							Environment-QOL	Baseline: 2·75 (SD: 0·69); Follow-up: 3·66 (SD: 0·67); p = 0·001
							Diagnosed with a mental illness *(people)*	Baseline: 5; Follow-up: 8; p = 0·38*favours control*
Kessler 2014[29]	RCT	USA	4604 Low income families living in assisted housing	Voucher to move to a low-poverty area or unrestricted moving voucher vs no voucher	The low poverty voucher group received counseling	120–180 months	Major depressive disorder: *Low Poverty voucher group*	Boys: OR 2·2 (95% CI 1·2–3·9); p = 0·03*favours control* Girls: OR 0·6 (95% CI: 0·3–1); p = 0·06*favours control* Combined: OR 1 (95%CI: 0·6–1·4); p = 0·84*favours control*
							Panic disorder: *Low Poverty voucher group*	Combined: OR 0·7 (95%CI: 0·4–1·1); p = 0·17*favours control*
							Posttraumatic stress disorder: *Low Poverty voucher group*	Boys: OR 3·4 (95% CI: 1·6–7·4); p = 0·007*favours control* Girls: OR 1·2 (95% CI: 0·8–2·1); p = 0·4*favours control* Combined: OR 1·8 (95%CI: 1·2–2·7); p = 0·03*favouring control*
							Oppositional-defiant disorder: *Low Poverty voucher group*	Combined: OR 0·7 (95%CI: 0·5–1·1); p = 0·17*favours control*
							Intermittent explosive disorder: *Low Poverty voucher group*	Combined: OR 0·8 (95%CI: 0·6–1); p = 0·13*favours control*
							Conduct disorder: *Low Poverty voucher group*	Boys: OR 3·1 (95% CI: 1·7–5·8); p<0·001*favours control* Girls: OR 0·5 (95% CI: 0·2–1·4); p = 0·2*favours control* Combined: OR 1·6 (95%CI: 1–2·6); p = 0·13*favours control*
							Major depressive disorder: *Traditional voucher group*	Boys: OR 1·7 (95% CI: 0·9–3·4); p = 0·23*favours control* Girls: OR 0·6 (95% CI: 0·3–0·9); p = 0·06*favours control* Combined: OR 0·9 (95%CI: 0·6–1·3); p = 0·7*favours control*
							Panic disorder: *Traditional voucher group*	Combined: OR 0·9 (95%CI: 0·5–1·5); p = 0·7*favours control*
							Posttraumatic stress disorder: *Traditional voucher group*	Boys: OR 2·7 (95% CI: 1·2–5·8); p = 0·05*favours control* Girls: OR 0·7 (95% CI: 0·3–1·2); p = 0·33*favours control* Combined: OR 1·1(95%CI: 0·7–1·8); p = 0·7*favours control*
							Oppositional-defiant disorder: *Traditional voucher group*	Combined: OR 1·1 (95%CI: 0·8–1·5); p = 0·7*favours control*
							Intermittent explosive disorder: *Traditional voucher group*	Combined: OR 0·9 (95%CI: 0·7–1·2); p = 0·7*favours control*
							Conduct disorder: *Traditional voucher group*	Boys: OR 2 (95% CI: 0·8–5·1); p = 0·23*favours control* Girls: OR 0·1 (95% CI: 0–0·4); p = 0·02 Combined: OR 0·9 (95%CI: 0·5–1·7); p = 0·7*favours control*
Aubry 2015[30]	RCT	Canada	950 High-need homeless adults with severe mental illness	Housing First vs treatment as usual	Participants in both groups had additional counseling and resources available	12 months	Stable housing,	OR 6·35; covariate adjusted difference 42% (95% CI: 36%-48%); p<0·001
							Quality of Life *(QOL)*	Mean change 7·27 (95%CI: 3·84–10·69); p<0·001
							Severity of psychiatric symptoms	Mean change -0·54 (95%CI: -2·26–1·17)*favours control*
							Community functioning	Mean change 1·81 (95%CI: 0·65–2·98); p = 0·003
Kirst 2015[31]	RCT	Canada	575 Homeless adults with serious mental illness in Toronto	Housing First vs treatment as usual	Participants in both groups had additional counseling and resources available	24 months	Substance misuse *(GAIN SS)*	IRR 0·86 (95%CI: 0·65–1·13)*favours control*
							Alcohol problems in 30 days	IRR 0·46 (95%CI: 0·23–0·91); p<0·05
							Drug problems on 30 days	IRR 0·66 (95%CI: 0·23–0·9)*favours control*
Somers 2015[32]	2 concurrent RCT's	Canada	497 Homeless adults with serious mental illness	Housing First vs treatment as usual	Participants in both groups had additional counseling and resources available	24 months	Percent of time stably housed moderate need-Intensive Case Management (ICM)	Intervention: 73% (SD:26·2); Control: 24·4% (SD: 27·3)*unknown significance*
							Daily substance use moderate need ICM	AOR 0·78 (95%CI: 0·37–1·63)*favours control*
Stergiopoulos 2015[33]	RCT	Canada	378 Homeless adults with serious mental illness	Housing First vs treatment as usual	Participants in both groups had additional counseling and resources available	24 months	Time in stable residence	Intervention: 75·1% (95% CI: 70·5–79·7); Control: 39·3% (95% CI: 34·3–44·2)
							Health status *(EQ5D-VAS)*	Change in mean difference -1·25 (95%CI: -6·96–4·46); p = 0·668*favours control*
							Substance use problem severity *(GAIN-SS)*	Change in mean difference 0·91 (95%CI: 0·65–1·28); p = 0·583*favours control*
							Physical community integration *(CIS-PHYS)*	Change in mean difference 1 (95%CI: 0·84–1·2); p = 0·959*favours control*
							Psychological community integration *(CIS-PSYCH)*	Change in mean difference 0·4 (95%CI: -0·58–1·38); p = 0·419*favours control*
							Quality of life *(QoLI)*	Change in mean difference 1·12 (95%CI: -3·81–6·06); p = 0·656*favours control*
Woodhall-melink 2015[34]	RCT	Canada	575 Homeless adults with serious mental illness	Housing First vs treatment as usual	Participants in both groups had additional counseling and resources available	24 months	BMI moderate needs:	B 0·00063; p = 0·99*favours control*
							Waist circumference- moderate needs	β 1·01; p = 0·52*favours control*
							BMI high needs:	B 0·91; p = 0·34*favours control*
							Waist circumference- high needs	β 2·1; p = 0·64*favours control*
Kozloff 2016[35]	RCT	Canada	156 Homeless youth with serious mental illness	Housing First vs treatment as usual	Participants in both groups had additional counseling and resources available	24 months	Days in stable housing:	Adjusted mean difference 34% (95%CI: 24–45); p = <0·001
							Number of arrests	Difference or ratio of changes from baseline (24 months) 0·67 (95%CI: 0·22–2·07); p = 0·39*favours control*
							Health *(EQ-5D)*	Difference or ratio of changes from baseline (24 months) 2·81 (95%CI: -6·36–11·97); p = 0·36*favours control*
							QOLI-20	Difference or ratio of changes from baseline (24 months) 7·29 (95%CI: -1·61–16·18); p = 0·17*favours control*
							MCAS	Difference or ratio of changes from baseline (24 months) 0·25 (95%CI: -2·79–3·28); p = 0·49*favours control*
							Community integration *(CIS)*	Difference or ratio of changes from baseline (24 months) 0·49 (95%CI: -0·99–1·98); p = 0·84*favours control*
							Recovery Assessment Scale *(RAS)*	Difference or ratio of changes from baseline (24 months) 1·8 (95%CI:-3·33–6·93); p = 0·49*favours control*
							Physical health *(SF-12)*	Difference or ratio of changes from baseline (24 months) 1·46 (95%CI:-2·83–5·74); p = 0·51*favours control*
							Mental health *(SF-12)*	Difference or ratio of changes from baseline (24 months) -0·78 (95%CI:-6·74–5·18); p = 0·59*favours control*
							Colorado Symptom Index *(CSI)*	Difference or ratio of changes from baseline (24 months) -0·05 (95%CI: -5·1–5); p = 0·84*favours control*
							GAIN-SPS	Difference or ratio of changes from baseline (24 months) 0·84 (95%CI: 0·51–1·38); p = 0·55*favours control*
							Victim of violent robbery, physical, or sexual assault	Difference or ratio of changes from baseline (24 months) 1·4 (95%CI: 0·55–3·57); p = 0·14*favours control*
Stergiopoulos 2016[36]	Pragmatic RCT	Canada	237 Moderate needs homeless adults with mental illness	Housing First vs treatment as usual	Participants in both groups had additional counseling and resources available	24 months	Participants housed	Intervention 75% (95%CI: 70–81); Control 41% (95%CI: 35–48)
							Number of arrests	Ratio of rate ratios 1·31 (95%CI: 0·37–4·62); p = 0·67*favours control*
							Number of days in past 30 experienced alcohol problems	Ratio of rate ratios 0·35 (95%CI: 0·12–1·02); p = 0·054*favours control*
							Number of days in the past 30 experienced drug problems	Ratio of rate ratios 0·58 (95%CI: 0·24–1·42); p = 0·23*favours control*
Aubry 2016[37]	RCT	Canada	950 Homeless adults with serious mental illness	Housing First with Assertive Community Treatment (ACT) vs treatment as usual	Participants in both groups had additional counseling and resources available	48 months	Time housed in previous 3 months	Intervention: baseline 10·78% (SD: 27·16); follow- up 72·6% (SD: 42·81); Control: baseline 8·64% (SD: 25·03); follow up 41·79% (SD: 47·61) *unknown significance*
							Days housed at final interview	Intervention: 280·74 (SD: 278·92); Control:115·33 (SD: 191·43) *unknown significance*
							Percent stable housing	Intervention follow up: 74% (95% CI: 69–78); Control follow up: 41% (95% CI: 35–46) *unknown significance*
							Length of stay *(days)*	Intervention follow up: 401·9 (95% CI: 372·2–430·2); Control follow up: 281·2 (95% CI: 251·2–318·6); P<0·001
							Quality of life *(QoLI-20)*	Intervention: baseline 73·99 (SD: 22·71); follow- up 89·38 (SD: 22·45); Control: baseline 72·39 (SD: 23·84); follow up 87·16 (SD: 22·57) *unknown significance*
							Physical integration	Intervention: baseline 1·95 (SD: 1·17); follow- up 1·81 (SD: 1·6); Control: baseline 1·97 (SD: 1·68); follow up 2 (SD: 1·74)*unknown significance*
							Psychological integration	Intervention: baseline 10·89 (SD: 3·79); follow- up 12·85 (SD: 3·34); Control: baseline 10·76 (SD: 3·87); follow up 12·75 (SD: 3·6)*unknown significance*
							Health status *(Eq-5D)*	Intervention: baseline 0·64 (SD: 0·24); follow- up 0·7 (SD: 0·24); Control: baseline 0·62 (SD: 0·24); follow up 0·72 (SD: 0·24)*unknown significance*
							Substance use *(GAIN)*	Intervention: baseline 1·93 (SD: 1·88); follow- up 1·47 (SD: 1·78); Control: baseline 1·95 (SD: 1·89); follow up 1·31 (SD: 1·73)*unknown significance*
Collins 2016[38]	Quasi-experiment	USA	134 Chronically homeless adults with alcohol problems	Before move-in to Housing First vs 2 years after move-in	Participants in both groups had additional counseling and resources available	24 months	Clinical significance of suicidal ideation	OR 0·33 (SE 0·09); p<0·001
							Intent to die by suicide	OR 0·45 (SE 0·18); p = 0·046
Somers 2017[39]	Randomized trial	Canada	297 Homeless adults with serious mental illness	Housing First vs treatment as usual	Participants in both groups had additional counseling and resources available	24 months	Severity of disability (MCAS)	Combined: p<0·001
							Community integration on physical subscale	Combined: p = 0·002
							Community integration psychological subscales	Combined: p<0·001
							Psychiatric symptom severity	Combined: p = 0·145*favours control*
							Overall health	Combined: p = 0·444*favours control*
							Food security	Combined: p = 0·079*favours control*
							Substance use problems	Combined: P = 0·486*favours control*
							Quality of life	Combined: p = 0·22*favours control*
							Recovery assessment	Combined: p = 0·0025
O’Campo 2017[40]	RCT	Canada	2148 Homeless adults with serious mental illness	Housing First vs treatment as usual	Participants in both groups had additional counseling and resources available	24 months	Homelessness duration ≥ 3 years moderate needs	Unadjusted OR 0·66 (95%CI: 0·52–0·84); p<0·01
							Community functioning variable MCAS total score moderate needs *(lower scores are associated with poorer functioning)*	Unadjusted OR 1·12(95%CI:1·02–1·24); p = 0·02
							CSI total score ≥ 30 moderate needs	Unadjusted OR 0·41 (95%CI:0·3–0·56); p = <0·01
							Days in the past month experienced alcohol problems moderate needs	Unadjusted OR 0·96 (95%CI:0·95–0·98); p = <0·01
							Days in the past month experienced drug problems moderate needs	Unadjusted OR 0·97 (95%CI:0·96–0·98); p = <0·01
							Physical health variables: Ulcer; *moderate needs*	Unadjusted OR 0·55 (95%CI:0·38–0·79); p = <0·01
							Physical health variables: bowel problems; *moderate needs*	Unadjusted OR 0·85 (95%CI:0·58–1·25); p = 0·41*favours control*
							Physical health variables: high blood pressure; *moderate needs*	Unadjusted OR 1·12 (95%CI:0·84–1·48); p = 0·43*favours control*
							Physical health variables; diabetes: *moderate needs*	Unadjusted OR 1·03 (95%CI:0·67–1·57); p = 0·9*favours control*
							Number of times participants achieved high or marginal food security- moderate needs Montreal	Rate ratio 1·02 (95%CI: 0·81–1·29); p = 0·84*favours control*
							Number of times participants achieved high or marginal food security: *moderate needs Toronto*	Rate ratio 0·98 (95%CI: 0·8–1·2); p = 0·84*favours control*
							Number of times participants achieved high or marginal food security: *moderate needs Winnipeg*	Rate ratio 1·12 (95%CI: 0·84–1·48); p = 0·44*favours control*
							Number of times participants achieved high or marginal food security: *moderate needs Vancouver*	Rate ratio 1·02 (95%CI: 0·8–1·3); p = 0·9*favours control*
							Homelessness duration ≥ 3 years high needs	Unadjusted OR 0·99 (95%CI: 0·76–1·31); p = 0·98*favours control*
							Community functioning variable: *high needs (MCAS)*	Unadjusted OR 0·88 (95%CI: 0·8–0·97); p = 0·01*favours control*
							CSI total score ≥ 30: *high needs*	Unadjusted OR 0·35 (95%CI: 0·24–0·49); p = <0·01
							Days in the past month experienced alcohol problems: *high needs*	Unadjusted OR 0·98 (95%CI: 0·96–0·99); p = 0·02
							Days in the past month experienced drug problems: *high needs*	Unadjusted OR 0·97 (95%CI: 0·95–0·98); p = <0·01
							Physical health variables: Ulcer; *high needs*	Unadjusted OR 0·56 (95%CI: 0·37–0·85); p = <0·01
							Physical health variables: bowel problems; *high needs*	Unadjusted OR 0·73 (95%CI: 0·47–1·14); p = 0·17*favours control*
							Physical health variables: high blood pressure; *high needs*	Unadjusted OR 0·65 (95%CI: 0·47–0·92); p = 0·01
							Physical health variables: diabetes; *high needs*	Unadjusted OR 0·74(95%CI: 0·47–1·17); p = 0·2*favours control*
							Number of times participants achieved high or marginal food security: *high needs Moncton*	Rate ratio 1·42 (95%CI: 1·04–1·95); p = 0·03
							Number of times participants achieved high or marginal food security: *high needs Montreal*	Rate ratio 0·89 (95%CI: 0·68–1·16); p = 0·38*favours control*
							Number of times participants achieved high or marginal food security: *high needs Toronto*	Rate ratio 1·48 (95%CI: 1·11–1·97); p<0·01
							Number of times participants achieved high or marginal food security: *high needs Winnipeg*	Rate ratio 0·81 (95%CI: 0·55–1·18); p = 0·27*favours control*
							Number of times participants achieved high or marginal food security: *high needs Vancouver*	Rate ratio 1·22 (95%CI: 0·95–1·56); p = 0·12*favours control*

*Results favor the intervention unless indicated otherwise

#### Food

There were 307 583 participants in the 17 food studies (Table 2). Food studies were conducted in USA (11 studies), Norway (two studies), Mexico (one study), Colombia (one study), New Zealand (one study), Ukraine (one study). One study (5·9%)involved a co-intervention consisting of nutrition and education counselling. [41] The most commonly measured health outcome was Body Mass Index (BMI) measured in 12studies (70·6%). The study durations ranged from four to 96 months. Food studies reported a total of 73 outcomes, of which 28 were statistically significant, 41 were not significant, and significance was unknown for four outcomes. Of the 28 statistically significant outcomes, 22 outcomes (from eight different studies) favoured the intervention, and six outcomes (from three different studies) favoured the control group.

**Table 2:** Characteristics of included food studies (N = 17).

Study	Study type	Country	Participants	Intervention vs. Comparison	Co-intervention	Time	Health Outcome	Results*
Murphy 1998[42]	Cross sectional and longitudinal observations	USA	169 Elementary school students	School breakfast program vs no school breakfast program	NR	4 months	Depression *(the children’s depression inventory scale)*	Intervention: baseline 3·4; follow up 4·2; Control: baseline 7·9; follow up 6·8 p < 0·01
							The revised children's manifest anxiety scale	Intervention: baseline 7·2; follow up 7·3; Control: baseline 11·4; follow up 7·2*favours control*
							Pediatric symptom checklist	Intervention: baseline 13·9; follow up 14·7; Control: baseline 18·9; follow up 17·2*favours control*
Gibson 2003[43]	Cohort	USA	6731 Low income adults	Current Food Stamp Program (FSP) participation vs no current FSP participation	NR	NR	Obese *(percent)*	Follow up: Intervention 29·7; Control 19·8; p<0·05*favours control*
							Overweight but not obese *(percent)*	Follow up: Intervention 26·9; Control 25·6*favours control*
							Underweight *(percent)*	Follow up: Intervention 2·5; control 2·8*favours control*
							BMI	Follow up: Intervention 27·6 (SEM 0·095); control 25·8 (SEM 0·064); p<0·05*favours control*
Gibson 2004[44]	Cohort	USA	7843 Children	Current Food Stamp Program (FSP) participation vs no current FSP participation	NR	NR	Overweight boys *(percent)*	Follow-up: intervention 16·8; control 17·3*favours control*
							BMI girls	Follow-up: intervention 19·11 (SEM 0·09); control 19·56 (SEM 0·052); p<0·1*unknown significance*
							Overweight girls	Follow-up: intervention 18 control 14·9; p<0·1 *unknown significance*
							BMI girls	Follow-up: intervention 19·68 (SEM 0·101); control 19·65 (SEM 0·071)*favours control*
Ramirez-lopez 2005[45]	A quasi-experimental, longitudinal prospective study	Mexico	610 School children	School breakfast program vs no school breakfast program	NR	NR	BMI	Intervention: baseline 17·1 (SD: 0·1); follow up 17·2 (SD: 0·1); Control: baseline 17 (SD: 0·2); follow up 16·9 (SD: 0·2)*favours control*
							Body fat *(percent)*	Intervention: baseline 29·5 (SD: 0·1); follow up 29·3 (SD: 0·1); Control: baseline 29·5 (SD: 0·2); follow up 29 (SD: 0·2)*favours control*
							Cholesterol *(mg/dl)*	Intervention: baseline 149·4 (95%CI: 148·3–157·4); follow up 147·7 (95%CI: 146·1–155·4); Control: baseline 149·1 (95%CI: 145·5–160·7); follow up 148·1 (95%CI: 144·3–157·6) P<0·05
							Triglycerides *(mg/dl)*	Intervention: baseline 55·1 (95%CI: 56·8–64·7); follow up 53·5 (95%CI: 54·8–62·3); p > 0·05 Control: baseline 58·6 (95%CI: 58·6–73·1); follow up 55·8 (95%CI: 55·7–70·2); p > 0·05*favours control*
							glucose fasting *(mg/dl)*	Intervention: baseline 84·1 (95%CI: 83·4–85·1); follow up 87·4 (95%CI: 86·7–88·5); p > 0·05 Control: baseline 85·4 (95%CI: 84·2–87·2); follow up 88·4 (95%CI: 87·3–90); p > 0·05*favours control*
Lee 2007[41]	Retrospective longitudinal study	USA	252, 246 Children in Illinois	Participant in food stamps, women infants and children (WIC) program vs non participants	WIC includes nutrition education and counseling	60 months	Abuse	mean of outcomes 0·024; p<0·05
							Neglect	mean of outcomes 0·023; p<0·05
							Anemea	mean of outcomes 0·103; p<0·05
							Failure to thrive	mean of outcomes 0·033; p<0·05
							Nutritional deficiency	mean of outcomes 0·002; p<0·05
Gleason 2009[46]	Cross sectional	USA	2228 School aged children	School breakfast or school lunch programs vs no food program	NR	NR	BMI: *school breakfast program*	coefficient from a linear regression model -0·149; p<0·05
							Overweight or obese status: *school breakfast program*	coefficient from a linear regression model -0·069 *favours control*
							Obese: *school breakfast program*	coefficient from a linear regression model -0·09*favours control*
							BMI: *school lunch program*	coefficient from a linear regression model 0·043*favours control*
							overweight or obese: *school lunch program*	coefficient from a linear regression model 0·046*favours control*
							Obese: *school lunch program*	coefficient from a linear regression model -0·003*favours control*
Arsenault 2009[47]	Observational	Colombia	3202 Children enrolled in the public primary school system age 5–12	School snack vs no school snack	NR	5 months	Hemoglobin,	Mean change 1 (95% CI: 0–2)*favours control*
							Plasma ferritin	Mean change 1·8 (95% CI: -0·1–3·7)*favours control*
							Plasma vitamin B-12,	Mean change 17 (95% CI: 9–25); p<0·0001
							Erythrocyte folate	Mean change -1 (95% CI: -26-23)*favours control*
							Height-for-age Z-score	Mean change 0·04 (95% CI: 0·02–0·05); p = 0·001
							BMI-for-age Z-scores	Mean change 0·02 (95% CI: -0·01–0·05)*favours control*
							Fever *(rate of days/child year)*	Unadjusted RR 0·63 (95% CI: 0·59–0·68); p = 0·0003
							Cough with fever *(rate of days/child year)*	Unadjusted RR 0·56 (95% CI: 0·50–0·62); p<0·0001
							Diarrhoea *(rate of days/child year)*	Unadjusted RR 0·68 (95% CI: 0·63–0·73); p = 0·03
							Diarrhoea with vomiting *(rate of days/child year)*	Unadjusted RR 0·63 (95% CI: 0·52–0·75); p = 0·0007
Ask 2010[48]	Controlled intervention	Norway	150 School students	Free school lunch vs no free school lunch	NR	4 months	Male BMI	Intervention: baseline 20·7 (SD: 3·1); follow up 21·3 (SD: 3·3) Control: baseline 20·8 (SD: 2·9); follow up 21·2 (SD: 3·1) p = 0·949*favours control*
							Female BMI	Intervention: baseline 20·5 (SD: 3·5); follow up 20·7 (SD: 3·4) Control: baseline 20·2 (SD: 2·8); follow up 20·5 (SD: 2·5) p = 0·725*favours control*
NiMhurchu 2010[49]	Step wedge cluster RCT	New Zealand	424 School age student	Free school breakfast vs no free breakfast	NR	12 months	Food security *(study child)*	OR 0·92 (95%CI: 0·7–1·22); p = 0·55*favours control*
							Food security *(all children in household)*	OR 0·89 (95%CI: 0·67–1·18); p = 0·43*favours control*
Chen 2011[50]	Cohort	USA	1723 Low income women	Food stamp participant vs non-participant	NR	NR	BMI	Coefficient 0·202 (SE: 0·086); p = 0·1*favours control*
							Obesity	Coefficient 0·013 (SE: 0·0009)*favours control*
Leung 2011[51]	A cross-sectional analysis of the 2007 Adult California Health Interview Survey	USA	7741 Adults in public assistance programs	People participating in food assistance programs vs non- participants	NR	NR	SNAP participants BMI	Adjusted difference 1·08 (95%CI: -0·5–2·22); p = 0·06*favours control*
							SNAP participants obesity *(BMI* ***≥*** *to 30*.*0kg/m2)*	Adjusted prevalence ratio 1·3 (95%CI: 1·06–1·59); p = 0·01*favours control*
							SSI participants BMI	Adjusted difference 1·83 (95%CI: 0·89–2·78); p<0·0001*favours control*
							SSI participants obesity *(BMI* ***≥*** *to 30*.*0kg/m2)*	Adjusted prevalence ratio 1·5 (95%CI: 1·27–1·77); p<0·0001*favours control*
							Calworks participants BMI	Adjusted difference 0·16 (95%CI: -1·07–1·4)*favours control*
							Calworks participants obesity *(BMI* ***≥*** *to 30*.*0kg/m2)*	Adjusted prevalence ratio 0·84 (95%CI: 0·66–1·07)*favours control*
Jilcott 2011[52]	Cross sectional study: analyzed data from the 2005–2006 National Health and Nutrition Examination Survey	USA	945 Food stamp eligible adults	Received food stamps vs no food stamps	NR	NR	BMI:	Intervention follow up: 30·5 (95% CI: 28·9–32·1) Control follow up: 28·3 (95% CI: 27·5–29·2) P = 0·01*favouring control*
							Waist circumference	Intervention follow up: 99·4 (95% CI: 96·1–102·6) Control follow up: 96·3 (95% CI: 94·2–98·4) P = 0·06*favours control*
Nicholas 2011[53]	Analyze data from the Health and Retirement Study (HRS), a nationally representative, longitudinal survey of older Americans	USA	558 Diabetic older adults	Received food stamps vs no food stamps	NR	NR	Food insufficient	Intervention: 0·27 (SD: 0·45) Control: 0·16 (SD: 0·36)*favours control*
							HbA1c	Intervention: 7·22 (SD: 1·35) Control: 7·11 (SD: 1·5)*favours control*
Schmeiser 2012[54]	Retrospective longitudinal study	USA	16553 Low- income children	Participated in Supplemental nutrition assistance program (SNAP) vs non-participants	NR	NR	BMI percentile girls	Number of past 60 months participating in SNAP (IV) Individual fixed- effects State fixed-effects: -0·3723; p<0·01
							Overweight girls	Number of past 60 months participating in SNAP (IV) Individual fixed- effects State fixed-effects: -0·0034; p<0·1*favours control*
							Obese girls	Number of past 60 months participating in SNAP (IV) Individual fixed- effects State fixed-effects: -0·0011*favours control*
							BMI percentile boys	Number of past 60 months participating in SNAP (IV) Individual fixed- effects State fixed-effects: -0·5574; p<0·01
							Overweight boys	Number of past 60 months participating in SNAP (IV) Individual fixed- effects State fixed-effects: -0·0078; p<0·01
							Obese boys	Number of past 60 months participating in SNAP (IV) Individual fixed- effects State fixed-effects: -0·0041; p<0·01
Leung 2013[55]	Multistage cross- sectional survey	USA	5193 Low income children	Participated in Supplemental Nutrition Assistance Program (SNAP) vs non-participants	NR	NR	Number of children overweight	Age and gender adjusted OR 0·94 (95%CI: 0·7–1·28)*favours control*
							Number of obese children	Age and gender adjusted OR 1·31 (95%CI: 0·91–1·89)*favours control*
Bere 2014[56]	Cluster randomized trial	Norway	320 Children: 10- to 12-year-old children from 2 Norwegian counties	Free fruit vs no free fruit	NR	96 months	BMI	Follow up: intervention 22·7 (95% CI: 22–23·4) Control 23.2 (95% CI: 22·6–23·8) p = 0·31*favours control*
							Percent overweight	Follow up: intervention 15 (95% CI: 8–21) Control 25 (95% CI: 19–31) p = 0·04
McMahon 2015[57]	Quasi-experimental regression discontinuity analysis	Ukraine	947 Children residing in the contaminated district after Chernobyl	3 Free meals vs 2 free meals (uses same sample group for both intervention and control at different times)	NR	NR	Individual whole body content of 137 Cesium adjusted for body weight *(Bq/m2)*	Spearman r = 0·26; p<0·001
							Unspecified anemia *(prevalence ratio)*	Follow up: three meals 0·57 (95%CI: 0·48–0·67); Two meals 1·31 (95%CI: 1·11–1·57) p<0·0001
							Allergy *(prevalence ratio)*	Follow up: three meals 1·41 (95%CI: 0·84–1·93); Two meals 1·26 (95%CI: 0·82–1·93); p = 0·72*favours control*
							Atopic dermatitis *(prevalence ratio)*	Follow up: three meals 1·22 (95%CI: 0·69–2·14); Two meals 1·02 (95%CI: 0·58–1·82); p = 0·52*favours control*
							Bronchitis *(prevalence ratio)*	Follow up: three meals 1·09 (95%CI: 0·81–1·48); Two meals 1·24 (95%CI: 0·81–1·9); p = 0·43*favours control*
							Common cold *(prevalence ratio)*	Follow up: three meals 1·27 (95%CI: 0·87–1·84); Two meals 2·32 (95%CI: 1·79–3); p = 0·01
							Lymph node enlargement *(prevalence ratio)*	Follow up three meals 1·01 (95%CI: 0·92–1·11); Two meals 1·07 (95%CI: 0·93–2·23); p = 0·49*favours control*
							Chronic tonsillitis/adenoiditis *(prevalence ratio)*	Follow up: three meals 0·91 (95%CI: 0·86–0·96); Two meals 0·93 (95%CI: 0·84–1·03); p = 0·52*favours control*
							Hemoglobin *(g/dL)*	3 meals: beginning (1993): 12·14 (12·05–12·22) end (1995): 12·63 (12·56–12·71) 2 meals: beginning (1996): 12·46 (12·39–12·52) end (1998): 12·72 (12·66–12·79) *unknown significance*
							BMI kg/m2	3 meals: beginning (1993): 17·22 (16·99–17·44) end (1995): 17·45 (17·27–17·63) 2 meals: beginning (1996): 17·67 (17·50–17·83) end (1998): 17·78 (17·61–17·94) *unknown significance*

*Results favor the intervention unless indicated otherwise

#### Hygiene/Water sanitation

There were 10 504 participants in the six hygiene or water sanitation studies (the household was the unit of analysis in two studies) (Table 3). The free goods distributed were toothbrushes and toothpaste (two studies), a drinking water disinfectant (two studies), and free soap (two studies). The studies were conducted in India (three studies), England (one study), Pakistan (one study), and Israel (one study). Three studies (50%) involved a co-intervention which consisted of social marketing, and educational campaigns. [58–60] The most common outcomes were diarrhoea prevalence in three studies (50%); infection prevalence in two studies (33·3%); and prevalence of dental carries reported in two studies (33·3%). The study durations ranged from nine months to 60 months. These studies reported a total of 34 outcomes, of which 15 were statistically significant, 11 were not significant, and significance was unknown for eight outcomes. All of the 15statistically significant outcomes (from three different studies) favoured the intervention.

**Table 3:** Characteristics of included hygiene/water sanitation studies (N = 6).

Study	Study type	Country	Participants	Intervention vs Comparison	Co-intervention	Time	Health Outcome	Results*
Davies 2002[58]	RCT	England	3731 Children from the age of 12 months to 5·5 years	Free fluoride toothpaste vs no free toothpaste	A leaflet was included with the packages	60 months	Decay-missing, and filled teeth index,	Mean change 16%; p = 0·05
							Caries	Mean change 8%; p = 0·001
Luby 2006[61]	Cluster RCT	Pakistan	1337 Households in squatter settlements	10 Neighborhoods received bleach, 9 neighborhoods received supplies for hand washing, 9 neighborhoods received flocculant- disinfectant, 10 neighborhoods received flocculant- disinfectant plus hand washing, 9 neighborhoods were control	NR	9 months	Diarrhoea daily longitudinal prevalence: *bleach water treatment*	difference from control -55% (95%CI: -17- -80)
							Diarrhoea daily longitudinal prevalence: *soap and hand washing promotion*	difference from control -51% (95%CI: -12- -76)
							Diarrhoea daily longitudinal prevalence flocculent: *disinfectant plus soap*	difference from control -64% (95%CI: -29- -90)
							Diarrhoea daily longitudinal prevalence: *flocculent- disinfectant water treatment*	difference from control -55% (95%CI: -18 - -80)
Livny 2007[62]	Cross-sectional study	Israel	1500 infants	Free tooth brushes and toothpaste vs no free good	NR	48 months	0 times brushed in the last 48 hours *(percent of children with caries)*	intervention = 12·8; control = 24 *unknown significance*
							1 times brushed in the last 48 hours *(percent of children with caries)*	intervention = 10·3; control = 13 *unknown significance*
							2 times brushed in the last 48 hours *(percent of children with caries)*	intervention = 21·9; control = 12 *unknown significance*
							3 times brushed in the last 48 hours *(percent of children with caries)*	intervention = 17·9; control = 10 *unknown significance*
							4 times brushed in the last 48 hours *(percent of children with caries)*	intervention = 13·2; control = 7 *unknown significance*
Boisson2013[59]	RCT	India	2163 Households with children under 5	Free sodium dichloroisocyanurate tablets vs no free sodium dichloroisocyanurate tablets	Intervention included a promotional campaign and instructions on how to use tablets	13 months	Diarrhea *(longitudinal prevalence)*	Prevalence ratio 0·95 (95% CI: 0·79–1·13)*favours control*
							Weight-for-age-z scores	Follow up: Intervention: -1·586 Control: -1·589 *favours control*
Das 2013[63]	Cohort	India	93 Patients with filarial lymphoedema	Free limb hygiene kit vs before recieving kit	NR	12 months	Frequency of acute dermato-lymphangioadenitis: *grade 1 (per year)*	Baseline 2·4; follow up 0·8 *unknown significance*
							Frequency of acute dermato-lymphangioadenitis: *grade 2 (per year)*	Baseline 3·4; follow up 1·2 *unknown significance*
							Frequency of acute dermato-lymphangioadenitis: *Grade 3 (per year)*	Baseline 4·8; follow up 1·8 *unknown significance*
Nicholson 2014[60]	Cluster randomized controlled study	India	1680 Households of children (5 years) and their families *(the number of participants was not 100% clear)*	Free soap vs no soap	Included a social marketing program aimed to educate, motivate and reward children for hand washing	~10 months	Target children diarrhoea	Observed relative risk reduction 25·3% (95%CI: 36·6–2·3); p = 0·03
							Target children Acute respiratory infections	Observed relative risk reduction 14·9% (95%CI: 29·6–8·3) p = 0·001
							Children aged 5 and under (non-target) diarrhoea	Observed relative risk reduction 32·5% (95%CI: 41·1–3·8); p = 0·023
							Children aged 5 and under (non-target) Acute respiratory infection	Observed relative risk reduction 20·5% (95%CI: 29–8·1); p = 0·001
							Children aged 6–15 (non-Target) diarrhoea	Observed relative risk reduction 30% (95%CI: 38·7–6·6); p = 0·01
							Children aged 6–15 (non-Target) acute respiratory infection	Observed relative risk reduction 11·8% (95%CI:24·4–5·6); p = 0·003
							whole families diarrhoea	Observed relative risk reduction 30·7% (95%CI: 37·5–5·5); p = 0·013
							whole families acute respiratory infection	Observed relative risk reduction 13·9% (95%CI:23·1–6·5); p = <0·001
							Target children boils	Intervention: 2·87; Control: 3·06; p = 0·839*favours control*
							Target children ear infection	Intervention: 0·99; Control: 1·35; p = 0·114*favours control*
							Target children eye infection	Intervention: 0·38; Control: 0·7; p = <0·001
							Target children headache	Intervention: 0·67; Control: 0·88; p = 0·227*favours control*
							Target children vomiting	Intervention: 1·07; Control: 1·22; p = 0·719*favours control*
							Whole families boil	Intervention: 1·84; Control: 1·65; p = 0·062*favours control*
							Whole families ear infection	Intervention: 0·65; Control: 0·79; p = 0·379*favours control*
							Whole families eye infection	Intervention: 0·62; Control: 0·8; p = 0·788*favours control*
							Whole families headache	Intervention: 2·98; Control: 2·58; p = 0·12*favours control*
							Whole families vomiting	Intervention: 0·92; Control: 0·84; p = 0·073*favours control*

*Results favor the intervention unless indicated otherwise

#### Insecticide treated nets (ITN)

There were 7661 participants in five studies providing ITN (Table 4). The studies were conducted in Cameroon (two studies), Ghana (one study), Kenya (one study), and Nigeria (one study). Three studies (60%) involved a co-intervention consisting of additional medical care, a social marketing campaign and preventative sulfadoxine-pyrimethamine treatment. [64–66] The most common outcomes measured were parasitaemia in three studies (60%); anemia in two studies (33·3%); malaria in two studies (33·3%). Other outcomes included mortality and birth weight. The study durations ranged from four months to 36 months. Eleven outcomes were reported, of which three were statistically significant, and eight were not. Of the three statistically significant outcomes (from three different studies), all favoured the intervention.

**Table 4:** Characteristics of included mosquito nets studies (N = 5).

Study	Study type	Country	Participants	Intervention vs· Comparison	Co-intervention	Time	Health Outcome	Results*
Browne 2001[64]	RCT	Ghana	1961 Pregnant women with special focus on primigravidae and secundigravidae	Insecticide Treated Net vs no net	Women also received free emergency obstetric care if needed	11 months	Mild anemia:	OR 0·88 (95%CI: 0·7–1·09); p = 0·47*favours control*
							Severe anemia:	OR 0·8 (95%CI: 0·55–1·16); p = 0·62*favours control*
							Parasitaemia<1999/ μl	OR 0·89 (95%CI: 0·73–1·08); p = 0·56*favours control*
							Parasitaemia>1999/ μl:	OR 1·11 (95%CI: 0·93–1·33); p = 0·55*favours control*
							Birthweight 2000-2500g:	OR 0·87 (95%CI: 0·63–1·19) p = 0·25*favours control*
							Birthweight <2000g:	OR 0·8 (95%CI: 0·48–1·32); p = 0·26*favours control*
Fegan 2007[65]	Longitudinal	Kenya	3500 Children under 5 years old	With Insecticide Treated Net vs without Insecticide Treated Net (use)	Included a social marketing campaign	36 months	Mortality	Rate Ratio 0·56 (95%CI: 0·33–0·96); p = 0·04
Anyaehie 2011[67]	Longitudinal	Nigeria	990 Pregnant women, nursing mothers and children under 5	Before and after distribution of the nets	NR	6 months	Prevalence of malaria parasitemia *(%)*	p = 0·73*favours control*
Apinjoh 2015[68]	Observational	Cameroon	800 Rural and semi-urban residents who had been living in the community during the free Insecticide Treated Nets (ITN) distribution campaign	ITN use vs no ITN use	NR	5 months	Susceptibility to malaria Parasitemia for people who did not sleep under an ITN	Adjusted odds ratio 1·7 (CI 1·14–2·54); p = 0·009
Fokam 2016[66]	Cross-sectional	Cameroon	410 Pregnant women	ITN use vs no ITN use	Also studied the combined effects of ITN and intermittent preventative treatment sulfadoxine-pyrimethamine	4 months	Malaria prevalence *(number of people)*	X^2^ = 6·188; p = 0·103*favours control*
							Anemia prevalence *(number of people)*	X^2^ = 8·673; p = 0·034

*Results favor the intervention unless indicated otherwise

#### Safety equipment

Six studies provided free safety equipment including smoke alarms, hip protectors, mouth guards, and safety equipment for young children (e.g. stair gates and cupboard locks) (Table 5). We were unable to identify the total number of participants in these studies because some reports did not specify this information. The studies were conducted in England (two studies), USA (one study), Ireland (one study), Israel (one study) and Australia (one study). Five studies (83·3%) involved a co-intervention consisting of educational materials and sessions,[10, 69–71] as well as advice,[72] and one study offered stickers to promote the use of safety equipment.[71] The common outcome reported in all six studies was injury. Study duration ranged from six months to 72 months. Safety equipment studies reported a total of 23 outcomes, of which eight were statistically significant, 11 were not significant, and significance was unknown for four outcomes. Of the eight statistically significant outcomes, all eight outcomes (from three different studies) favoured the control and, according to the explanations provided in the articles, this may be been due to infrequent use of the safety equipment.[10, 71, 73]

**Table 5:** Characteristics of included safety equipment studies (N = 6).

Study	Study type	Country	Participants	Intervention vs· Comparison	Co-intervention	Time	Health Outcome	Results*
Mallonee 2000[70]	Community intervention trial- pre and post design	USA	9291 Homes in the Oklahoma city area	Free smoke alarm vs no free smoke alarm	Were given written educational material, and periodic fire alarm tests to ensure distributed alarms were functioning correctly	72 months	Injury rates per 100 residential fires	Intervention = baseline 5·02, follow up 1·2; Control = baseline 1·95, follow up 2·19*unknown significance*
							Injury rate per 100000 population	Intervention = baseline 15·35, follow up 2·96; Control = baseline 3·63, follow up 3·37 *unknown significance*
DiGuiseppi 2002[69]	Cluster RCT	England	Mean of 8191 primarily households including elderly people or children	Free smoke alarm vs no free smoke alarm	Smoke alarms were given with a fitting, educational brochures, and installation upon request	37 months	All injuries	Rate ratio 1·3 (95% CI 0·9–1·8)*favours control*
							Hospitalizations and deaths	Rate ratio 1·3 (95% CI 0·7–2·4)*favours control*
							Preventable injuries	Rate ratio 1·1 (95% CI 0·8–1·7)*favours control*
							Preventable hospitalizations and deaths	Rate ratio 1 (95% CI 0·5–1·9)*favours control*
O’Halloran 2004[71]	Cluster RCT	Ireland	Residents from 127 Nursing homes *(~4117 residents)*	Given hip protectors vs no hip protectors	A 1 hour information session was conducted with nursing home staff and support was given to nursing staff to implement this program, as well as posters and stickers promoting the use of hip protectors	18months	Number of hip fractures *(rate per 100 occupied beds)*	Unadjusted rate ratio 1·05 (95%CI: 0·76–1·45)*favours control*
							Number of pelvic fractures*(rate per 100 occupied beds)*	Unadjusted rate ratio 4·03 (95%CI: 1·51–10·74)*favours control*
							Number of injurious falls*(rate per 100 occupied beds)*	Unadjusted rate ratio 1·21 (95%CI: 0·79–1·83)*favours control*
Watson 2005[72]	RCT	England	3428 Families of children younger than 5	Intervention received free or low cost safety equipment *(Fitted stair gates*, *fire guards*, *smoke alarms*, *cupboard locks*, *and window locks)*vs usual care	Provided a consultation/advice	24 months	Child in family had a medically attended injury	OR 1·14 (95% CI: 0·98–1·5)*favours control*
							Abbreviated injury scale ≥2	OR 1·14 (95% CI: 0·76–1·71)*favours control*
							Minor injury severity score ≥2	OR 0·98 (95% CI: 0·75–1·27)*favours control*
Zadik 2009[73]	Retrospective study	Israel	Infantry units in the Israel Defense Forces *(630 participants)*	Intervention received boil an bite mouth guards vs control receiving none	NR	NR	Number of sports related oro-facial traumas	Intervention: 38/272; Control: 31/358; p<0·05*favours control*
							Dental fractures	Intervention: 25/272; Control: 17/358; p≤ 0·001*favours control*
							Dental luxations/subluxations	Intervention: 4/272; Control: 4/358*favours control*
							Lip laceration	Intervention: 16/272; Control: 7/358; p≤ 0·001*favours control*
							Chin laceration	Intervention: 8/272; Control: 5/358; p <0·05*favours control*
							Dislocation and/or pain of TMJ	Intervention: 6/272; Control: 1/358; p≤ 0·001*favours control*
							Fracture of mandible	Intervention: 0/272; Control: 1/358; p≤ 0·001*favours control*
Cameron 2011[10]	RCT	Australia	308 Older adults in the hospital	Free hip protector vs no free hip protector	There were three arms of the study: the control- who received a brochure about hip protectors, the no cost group- who were fitted with free hip protectors and the combined group- received free hip protectors and educational sessions about their use	6 months	Number of falls: hospital *(mean per participant)*	Intervention: 0·32; Control: 0·12; *X*^2^ = 9·114; p = 0·01*favours control*
							Number of fracture: hospital	Intervention: 5; Control: 1*unknown significance*
			171 Older adults in the community				Number of fall: community *(mean per participant)*	Intervention 0·28; Control: 0·13; *X*^2^ = 2·068; p = 0·356*favours control*
							Number of fractures: community	Intervention: 2; Control: 0*unknown significance*

*Results favor the intervention unless indicated otherwise

#### Miscellaneous

Five studies involved a miscellaneous set of outcomes (Table 6). The distributed free goods included glucometer test strips for diabetic patients, glucometers, sunscreen, bus passes, and a mobile phone. Three studies (60%) involved a co-intervention consisting of a glucometer (intervention was test strips),[74] educational material and counselling (for the glucometer study) [75] as well as an automated message and calling card to reach participants’ primary care physicians (for the mobile phone study) [76]. The outcomes measured included HbA1c, blood glucose, triglycerides, Low Density Lipoprotein (LDL-C), Body Mass Index (BMI), waist circumference, rate of sunburns, and mortality rate. The study durations ranged from two months to 12 months. These studies reported 13 outcomes, of which three were statistically significant, eight were not significant, and significance was unknown for two outcomes. All three statistically significant outcomes (from two different studies) favoured the intervention.

**Table 6:** Characteristics of included other studies (N = 5).

Study	Study type	Country	Participants	Intervention vs· Comparison	Co-intervention	Time	Health Outcome	Results*
Nyomba 2004[74]	RCT	Canada	62 Diabetics	Received test strips for their free glucometer vs no free test strips for free glucometer	Both groups received a free glucometer	12 months	HbAC1c	p = <0·002
							Random blood glucose measured at each doctor visit	p = <0·005
Nicol 2007[77]	Three-arm prospective randomized trial	France	364 People staying at beach resorts	Free sunscreen vs no free sunscreen	NR	2 months	Sunburn during the week in the free sunscreen group vs control	Intervention 29·9%; Control 46·8%*favours control*
							Sunburn during the week in the free new labelled sunscreen group vs control	Intervention 21·2%; Control 46·8%*favours control*
Webb 2012[78]	Longitudinal design	England	Elderly residents	Intervention received a free bus pass, control was not eligible	NR	NR	BMI	mean change: Intervention: 0·22 (95%CI: 0·15–0·28) Control: 0·6 (95%CI: 0·43–0·77)*unknown significance*
							Waist circumference	mean change: Intervention: 1·65 (95%CI: 1·47–1·83) Control: 2·17 (95%CI: 1·7–2·64)*unknown significance*
Guo 2014[75]	RCT	China	132 Low income with type 2 diabetes	Received glucometers vs no free glucometers	education materials and counseling were provided to all groups	6 months	HbA1c	Overall difference between groups based on one-way ANOVA = -0·13 (95% CI: -0·38- -0·12); p = 0·29*favours control*
							BMI	Overall difference between groups based on one-way ANOVA = 0·05 (95% CI: -0·34–0·44); p = 0·79*favours control*
							Triglycerides	Overall difference between groups based on one-way ANOVA = -0·14 (95% CI: -0·45–0·18); p = 0·39*favours control*
							LDL-C	Overall difference between groups based on one-way ANOVA = 0·01 (95% CI: -0·15–0·16); p = 0·92*favours control*
Lund 2014[76]	Cluster RCT	Zanzibar	2550 Pregnant women	Received mobile phone vs no free mobile phone	There was an automated short message component in addition to the intervention	NR	Still birth	Unadjusted odds ratio 0·62 (95%CI: 0·31–1·22)*favours control*
							Perinatal mortality rate	Unadjusted odds ratio 0·49 (95%CI: 0·27–0·9
							Neonatal mortality rate	Unadjusted odds ratio 0·85 (95%CI: 0·37–1·95)*favours control*

*Results favor the intervention unless indicated otherwise

### Results by health outcome

In addition to analyzing the results of studies categorized by type of free good distributed to participants, we combined results from the reviewed studies for the health outcomes of mortality and diarrhea because these two outcomes were reported in studies of different categories of goods.

#### Mortality

Mortality was reported as a health outcome in three studies of mosquito nets (one study), housing vouchers (one study), and mobile phones (one study) including 17 730 participants. The first study gave families with children under five an insecticide treated insect net in Kenya. The study found that receiving a mosquito net was a significant predictor of reduced mortality (rate ratio: 0·56; 95% confidence interval (CI): 0·33–0·96).[65] The second study gave a housing voucher to families of children living in public housing in the USA.[24] Receiving a housing voucher was not a significant predictor of mortality in any of the 3 categories; deaths from disease (p = 0·84), deaths by homicide (p = 0.81),and accidental deaths (p = 0·19).[24]The final study gave phones to pregnant women in Zanzibar. [76] Mortality was recorded in three ways: stillbirth (unadjusted odds ratio (UOR): 0·62; 95%CI: 0·31–1·22), perinatal mortality (UOR: 0·49; 95%CI: 0·27–0·90), and neonatal mortality (UOR: 0·85; 95%CI: 0·37–1·95). Receiving a free phone significantly reduced perinatal mortality. [76]

#### Diarrhea

Diarrhea was reported as a health outcome in four studies of food (one study), and hygiene and water sanitation (three studies), which included 8382 participants. The first study conducted in Pakistan included households in squatter settlements receiving either bleach, hand washing supplies, flocculant-disinfectant, or flocculant- disinfectant plus hand washing. [61] The authors concluded that receiving any of the free goods, as well as the intense community-based intervention, which included meetings and presentations to community leaders and residents about the importance of hygiene wand water contamination, reduced the daily longitudinal prevalence of diarrhoea; however, the level of statistical significance was not reported. [61]The second study, conducted in Colombia, gave primary school children a school snack. [47] The authors found that the rate of days per child year of diarrhoea (unadjusted rate ratio (URR):0·68; CI: 0·63–0·73), and diarrhoea with vomiting (URR: 0·63; CI: 0·52–0·75) were significantly reduced with the provision of a school snack.[47] The third study, conducted in India, gave children under the age of five sodium dichloroisocyanurate tablets.[59] The authors found that the longitudinal prevalence of diarrhoea for children given sodium dichloroisocyanurate tablets was not significantly different from the control (prevalence ratio: 0·95; CI: 0·79–1·13). [59]The final study, conducted in India, distributed soap to households with children under five, and outcomes were assessed for the target children, as well as their family, including siblings.[60] The authors reported significant relative risk reductions (RRR) in diarrhoea prevalence related to the provision of free soap among four groups: target children (RRR: 25·3%; CI 36·6–2·3); children aged five and under (non-target) (RRR: 32·5%; CI 41·1–3·8); children aged six-15 (non-target) (RRR: 30%; CI 38·7–6·6); and whole families (observed RRR 30·7%: CI 37·5–5·5). [60] As such, three of the four studies reported that diarrhoea was significantly reduced with the provision of free goods.

## Interpretation

The results of this systematic review provide evidence that free goods can improve health outcomes in certain circumstances, although there are also important gaps and limitations in the existing literature. Housing provision for people with serious mental health conditions in high-income countries and food provision to low-income children in high-income countries are supported by the largest number of studies. Of the 59 reviewed studies involving 379 932 participants (most were individuals but some were households) that examined the health effects of free goods, the most commonly studied free goods were housing (20 studies) and food (17 studies). Among the 268 total outcomes reported, the most commonly reported outcomes were housing retention in 12 housing studies and BMI in 12 food studies. Four RCTs were deemed to be unclear or at high risk of bias, and one non-RCT was rated as serious, critical or no information, in all risk of bias categories. Therefore, overall the studies were of medium to high quality in terms of bias. Among the studies included in this review, 80 health outcomes were statistically significant favouring the intervention, 19 health outcomes were statistically significant favouring the control, 141 health outcomes were not significant, and significance was unknown for 28 health outcomes.

The rationale underpinning how the provision of free tangible goods impacts health was typically not stated in the reviewed studies. However, we identify four related concepts that help us understand the rationale for providing free tangible goods. First, facilitating access to a good that is capable of promoting health should promote health unless there are unintended negative effects or implementation problems. We did in fact find some studies where those receiving a free good had worse health outcomes (e.g. hip protectors were associated with an increased risk of hip fractures).[71] Second, if poverty is defined, at least partially, as being unable to afford tangible goods (and services) in a market-based economy,[79] then studies examining the impact of free good provision on health describe the effect of poverty reduction on health. Findings from these studies could then be considered alongside studies of other interventions aimed at reducing poverty, such as a basic income as a complementary approach to reducing poverty.[12, 13] Third, the free provision of goods could be understood as “non-cash” income that is valued similar to its cash equivalent after being appropriately discounted.[6] Fourth, having certain tangible goods can be understood as fulfilling a basic human right (e.g. the right to adequate housing, the right to adequate nutrition and clean water).[80] The provision of such goods could be seen as achieving social justice and could have positive impacts not only for individuals but also for their communities.

## Comparison with prior studies

To the best of our knowledge this is the first systematic review to examine a wide range of free tangible goods and their effects on health. One recent systematic review and narrative analysis of 31 Housing First studies found mixed results for the impact of providing free housing for substance abuse and psychiatric symptoms, a clear benefit for housing stability, and a benefit for quality of life. These findings generally align well with ours. [81]

A number of studies have examined whether people who were given free goods use them or resell them. One such study conducted among pregnant women and households with young children in Uganda, for example, investigated this concept with the provision of free long-lasting insecticide treated mosquito nets. [82] This study assessed the willingness to pay for a mosquito net and willingness to sell a mosquito net given for free by simulating market exchanges. Seventy-three percent of people who received free nets were unwilling to accept the maximum price offered to part with even one of their nets. [82] Most people who were given free nets were not likely to resell their nets and in fact did use them for their intended purpose. [82]

Other studies have investigated using financial investments to complement health interventions and further improve health outcomes. A non-randomized controlled assessment from sub-Saharan Africa, in which simultaneous investments were made in agriculture, the environment, business development, education, infrastructure, and health in rural village sites with high baseline levels of poverty and under nutrition, found that mortality rates in young children decreased by 22% in study sites relative to baseline.[83] Reductions in poverty, food insecurity, stunting, and malaria parasitemia were also reported in study sites. [83]

## Strengths and limitations of our study

Due to the great variety of free goods with potential to impact health, the design of a search strategy was challenging and we may have inadvertently omitted some key search terms. The wide array of interventions and outcomes meant that we could not perform a meta-analysis of results. The broad approach allowed us to include an interesting array of studies of different free tangible goods. Some studies involved co-interventions (e.g. almost all housing studies involved other supports in addition to free housing) and this limits the ability to determine whether the free good or the co-intervention affected health outcomes. We also excluded many studies that provided free tangible goods, including clean needles, condoms, and baby cribs, but did not report a health outcome. The literature may be biased towards studies of items with a less certain benefits. In other words, researchers may have decided not to study certain goods which are very likely to be beneficial (e.g. condoms, clean needles) and some such studies may not be ethical (i.e. it may be difficult to study the free provision of an item that is very likely to be beneficial). Some of the Housing First studies were overlapping as different reports included some of the same participants and some of the same outcomes, so we attempted to strike a balance between not excluding results and not counting the same results twice.

## Conclusions and future work

Findings of this systematic review suggest that providing free tangible goods can promote health in certain circumstances. Additional high-quality studies of different goods are needed. Future work should also focus on the contexts in which free goods are most beneficial and explicitly state the theory or theories underpinning each study or intervention.

## Supporting information

S1 Checklist PRISMA checklist. (DOC)

S1 File Search strategy. (DOCX)

S1 Table Cochrane risk of bias assessment. (DOCX)

S2 Table ROBINS 1 risk of bias assessment. (DOCX)
