# Supplementary material for: Health outcomes related to the provision of free, tangible goods: A systematic review
Source: PLoS One. 2019 Mar 20;14(3):e0213845. doi: 10.1371/journal.pone.0213845 (PMC6426236; doi:10.1371/journal.pone.0213845)
Supplement: S1 File — (DOCX) [file pone.0213845.s002.docx]

**S1 File, Search strategy**

Searches were run using the following search terms: “first dollar”, “Free Apartment*”, “Free House*”, “Free Condominium*”, “Free Room*”, “Free Rent”, “Free housing”, “Free Food”, “Free Food Bank*”, “Free Groceries”, “Free Food Stamp*”, “Free Fruit*”, “Free Vegetables”, “Free Cooking Essentials”, “Free Oil”, “Free Meat”, “Free Meals”, “Free Baby Food”, “Free Milk”, “Free Oral Supplements”, “Free Vitamins”, “Free School Breakfast Program*”, “Free Seeds”, “Free Refrigerator*”, “Free Stove*”, “Free Bike*”, “Free Bus Voucher*”, “Free Train Voucher*”, “Free Subway Voucher*”, “Free Bus Pass*”, “Free Train Pass*”, “Free Subway Pass*”, “Free Bus Token*”, “Free Train Token*”, “Free Subway Token*”, “Free Bus Ticket*”, “Free Train Ticket*”, “Free Subway Ticket*”, “Free Bike Helmet*”, “Free Bicycle Helmet*”, “Free Beds”, “Free Chairs”, “Free Desks”, “Free Tables”, “Free Cribs”, “Free Books”, “Free Notebooks”, “Free School Supplies”, “Free Computers”, “Free Computer Access”, “Free Cellphone*”, “Free Mobile Phone*”, “Free Phone*”, “Free Calling Cards”, “Free Antiseptics”, “Free Bandage*”, “Free First Aid Kit*”, “Free Wheelchair*”, “Free Crutches”, “Free Canes”, “Free Air Cast Boot”, “Free Walkers”, “Free Glucometer*”, “Free Glucose Testing Strips”, “Free Lancets”, “Free Insulin Syringe*”, “Free Insulin Pump*”, “Free Pedometer*”, “Free Thermometers”, “Free Scales”, “Free Bathroom Grab Bars”, “Free Bath Chairs”, “Free CPAP Machine*”, “Free CPAP Machine Cleaning Kit*”, “Free Oxygen Tank*”, “Free Oxygen Mask*”, “Free Trach* Care Kit*”, “Free Trach* Cleaning Kit*”, “Free Clean Needle Exchange”, “Free Needle*”, “Free Overdose Reversal Kit*”, “, “Free Prosthetic*”, “Free Hearing Aids”, “Free Eye Glasses”, “Free Socks”, “Free Shoes”, “Free Anti-Slip Socks”, “Free Anti-Slip Slippers”, “Free Anti-Slip Pads”, “Free Compression Stockings”, “Free Medical Bracelets”, “Free Emergency Alert Device*”, “Free Diapers”, “Free Mittens”, “Free Jackets”, “Free Helmet*”, “Free Underwear”, “Free Soap”, “Free Shampoo”, “Free Lice Shampoo”, “Free Toothpaste”, “Free Toothbrush*”, “Free Floss”, “Free Detergent”, “Free Bathroom Cleaners”, “Free Disinfectant*”, “Free Baby Wipes”, “Free Water Filters”, “Free Water Purification Tablets”, “Free Sanitary Pads”, “Free Condom*”, “Free Pill Organizer*”, “Free Batteries”, “Free Physical Activity Tracker*”, “Free Sunscreen”, “Free Sports Equipment”, “Free Sports Gear”, “Free Personal Protective Equipment”, “Free PPE”, “Free Gloves”, “Free Boots”, “Free Masks”, “Free Eye Protection”, “Free Solar Panels”, “Free Back Up Generators”, “Free Insecticide-Treated Net*”, “Free Mosquito Net*”, “Free Insecticide-Treated Bednet*”, “Free Insecticide-Treated Bed Net*”, “Free Mosquito Bed Net*”, “Free Bednet*”, “Free Bed Net*”, “no charge”, “no cost”, “no expense”, “free of cost”, “free distribution”, “distributed free*”, “freely distributed”, “free baby box*”

Exclude “free radical”, “symptom free”, “free testosterone”, “free TSH”, “disease free”.
