# Supplementary material for: Health outcomes related to the provision of free, tangible goods: A systematic review
Source: PLoS One. 2019 Mar 20;14(3):e0213845. doi: 10.1371/journal.pone.0213845 (PMC6426236; doi:10.1371/journal.pone.0213845)
Supplement: S1 Table — (DOCX) [file pone.0213845.s003.docx]

**S1 Table, Cochrane risk of bias assessment**

| **Cochrane Risk of Bias (RCT)** | | | | | | | | |
| --- | --- | --- | --- | --- | --- | --- | --- | --- |
| **Author** | **Year** | **Random Sequence Generation** | **Allocation concealment** | **Blinding of Participants** | **Blinding of Outcome Assessment** | **Incomplete Outcome Data** | **Selective Reporting** | **Funding Source** |
| Aubry | 2016 | Low | High | High | Unclear | Low | Unclear | Unclear |
| Aubry | 2015 | Low | High | High | Unclear | low | Unclear | low |
| Bere | 2014 | Unclear | Unclear | High | Unclear | High | Unclear | Unclear |
| Boisson | 2013 | Low | Low | Low | Low | Unclear | Unclear | Unclear |
| Browne | 2001 | Unclear | Unclear | High | Unclear | High | Unclear | Unclear |
| Cameron | 2011 | Low | Low | High | Low | High | Unclear | Low |
| Davies | 2002 | Unclear | Unclear | High | Unclear | High | Unclear | High |
| DiGuiseppi | 2002 | Low | Low | High | Low | Unclear | Unclear | Low |
| Kessler | 2014 | Low | Low | High | Unclear | Low | Unclear | Unclear |
| Kirst | 2015 | Low | Low | High | High | Unclear | Unclear | Low |
| Kozloff | 2016 | Low | Low | High | High | Unclear | Unclear | Low |
| Luby | 2006 | Low | Unclear | High | High | Low | Unclear | Unclear |
| Lund | 2014 | Low | Unclear | High | High | Unclear | Unclear | Low |
| NiMhurchu | 2012 | Low | High | High | High | Low | Unclear | Low |
| Nicholson | 2014 | Low | Low | High | High | Unclear | Unclear | High |
| Nicol | 2007 | Unclear | Unclear | Low | Low | Unclear | Unclear | High |
| Nyomba | 2004 | Unclear | Unclear | Low | High | High | Unclear | High |
| O'campo | 2017 | Low | High | High | High | Unclear | Unclear | Low |
| O'Halloran | 2004 | Low | High | High | High | Unclear | Unclear | High |
| Palepu | 2013 | Unclear | Unclear | High | High | Low | Unclear | Unclear |
| Patterson | 2013 | Unclear | High | High | High | Low | Unclear | Low |
| Somers | 2015 | Low | High | High | High | Low | Unclear | Low |
| Somers | 2017 | Low | High | High | High | Unclear | Unclear | Unclear |
| Stefancic | 2007 | Unclear | Unclear | High | High | Unclear | Unclear | Unclear |
| Stergoipoulos | 2015 | Low | High | High | High | Low | Unclear | Low |
| Stergoipoulos | 2016 | Low | Low | High | High | Unclear | Unclear | Low |
| Tsemberis | 2004 | Unclear | Low | High | High | Low | Unclear | Unclear |
| Watson | 2005 | Low | Low | High | High | Low | Unclear | Low |
| Woodhall-Melnik1 | 2015 | Low | High | High | High | Unclear | Unclear | Low |
