# Supplementary material for: Health outcomes related to the provision of free, tangible goods: A systematic review
Source: PLoS One. 2019 Mar 20;14(3):e0213845. doi: 10.1371/journal.pone.0213845 (PMC6426236; doi:10.1371/journal.pone.0213845)
Supplement: S2 Table — (DOCX) [file pone.0213845.s004.docx]

**S2 Table, ROBINS 1 risk of bias assessment**

| **ROBINS 1 (observational)** | | | | | | | | |
| --- | --- | --- | --- | --- | --- | --- | --- | --- |
| **Author** | **Year** | **Bias due to confounding** | **Bias in selection of participants into the study** | **Bias in classification of interventions** | **Bias due to deviations from intended interventions** | **Bias due to missing data** | **Bias in measurement of outcomes** | **Bias in selection of the reported result** |
| Anyaehie | 2011 | Moderate | Moderate | No information | No information | No information | Low | No information |
| Apinjoh | 2015 | Moderate | Low | Critical | Moderate | No information | Low | No information |
| Arsenault | 2009 | Moderate | Critical | Low | Low | Moderate | Low | No information |
| Ask | 2010 | Serious | Low | Low | Moderate | Low | Moderate | No information |
| Bean | 2013 | Moderate | Low | No information | No information | Low | Low | No information |
| Chen | 2011 | Moderate | Low | Moderate | No information | No information | Low | No information |
| Collins | 2016 | Moderate | Serious | Low | Low | Low | Low | No information |
| Das | 2013 | Low | Low | Low | No information | Low | Low | No information |
| Fegan | 2007 | Moderate | Low | No information | No information | Low | Low | No information |
| Fokam | 2016 | Moderate | Low | Serious | No information | No information | Low | No information |
| Gibson | 2003 | Serious | Low | Moderate | No information | No information | Low | No information |
| Gibson | 2004 | Serious | Low | No information | No information | No information | Low | No information |
| Gleason | 2009 | Serious | Serious | Moderate | Moderate | Low | No information | No information |
| Guo | 2014 | Moderate | Low | Low | Moderate | Low | Low | No information |
| Jacob | 2013 | Moderate | Low | Low | Low | Moderate | Low | No information |
| Jilcott | 2011 | Moderate | Low | Low | No information | Low | Low | No information |
| Lee | 2007 | Serious | Low | Low | Serious | No information | Low | No information |
| Leung | 2013 | Moderate | Moderate | Low | Low | Moderate | Moderate | No information |
| Leung | 2011 | Moderate | Moderate | Low | Low | Moderate | Moderate | No information |
| Livny | 2007 | Moderate | Low | Moderate | No information | Serious | Low | No information |
| Mallonee | 2000 | Serious | Serious | No information | No information | No information | Low | No information |
| McMahon | 2015 | Moderate | Low | Low | No information | No information | No information | No information |
| Montgomery | 2013 | Moderate | No information | Low | Low | Low | No information | No information |
| Murphy | 1998 | Serious | Moderate | Critical | Moderate | Low | Serious | No information |
| Nicholas | 2011 | Serious | Critical | Critical | No information | No information | No information | No information |
| Padgett | 2011 | Serious | Moderate | Low | Low | Low | Moderate | No information |
| Ramirez-Lopez | 2005 | Serious | No information | Low | No information | No information | No information | No information |
| Schmeiser | 2012 | Moderate | Serious | Moderate | no information | Low | Low | No information |
| Webb | 2012 | Critical | Critical | Serious | No information | Serious | Moderate | No information |
| Zadik | 2009 | Serious | Serious | Serious | No information | No information | Low | No information |
